# IgE responses to exogenous and endogenous allergens in atopic dermatitis patients under long‐term systemic cyclosporine A treatment

**DOI:** 10.1111/all.12711

**Published:** 2015-09-17

**Authors:** S. Lucae, P. Schmid‐Grendelmeier, B. Wüthrich, D. Kraft, R. Valenta, B. Linhart

**Affiliations:** ^1^ Division of ImmunopathologyDepartment of Pathophysiology and Allergy ResearchCenter for Pathophysiology, Infectiology and ImmunologyMedical University of ViennaViennaAustria; ^2^ Department of DermatologyAllergy UnitUniversity Hospital ZürichZürichSwitzerland; ^3^ Present address: Max Planck Institute of PsychiatryMunichGermany

**Keywords:** allergy, atopic dermatitis, cyclosporin A, IgE

## Abstract

Atopic dermatitis (AD) patients mount IgE antibody responses to a variety of environmental allergens and also to autoantigens. We analyzed serum samples from four AD patients who had received oral cyclosporine A (CyA) treatment for up to 17 months regarding IgE autoreactivity to nitrocellulose‐blotted human epithelial cell extracts and IgE levels to environmental allergens by quantitative ImmunoCap measurements. Skin inflammation was assessed by SCORAD. During full‐dose treatment, a strong reduction in T‐cell‐mediated skin symptoms was observed which reappeared when CyA treatment was reduced or stopped. The intensity of IgE autoreactivity seemed to follow skin inflammation as it was reduced during full‐dose treatment and increased upon inflammation. Interestingly, IgE levels to exogenous allergens were boosted by allergen exposure, declined thereafter, and seemed to be unaffected by CyA. Our data thus indicate that allergen‐specific IgE production is boosted by allergen contact and cannot be reduced by CyA‐mediated T‐cell suppression.

Type I allergy is an IgE‐mediated hypersensitivity disease affecting almost 25% of the population in industrialized countries 1. Allergic sensitization occurs in genetically predisposed individuals early in childhood after allergen encounter which leads to class‐switching to IgE production, a process that depends on T‐cell help and production of Th2 cytokines 2, 3. The analysis of IgE reactivities to multiple micro‐arrayed allergen molecules in follow‐up serum samples obtained from children during the first years of life in birth cohort studies indicates that IgE sensitizations to new allergens become detectable during the first years of life indicating that the children expand their IgE reactivity profiles 4, 5. By contrast, IgE reactivity profiles in adult allergic patients remain stable and only allergen‐specific IgE levels change depending on allergen exposure 6, 7. Using different experimental models, evidence has been provided that the secondary IgE production in sensitized allergic subjects or animals does not require T‐cell help. For example, it has been demonstrated that primary allergic sensitization can be prevented by co‐stimulation blockade, whereas secondary IgE production is not affected in a murine model of grass pollen allergy 8. In a clinical study, it has been shown that only intact, IgE‐reactive allergens but not T‐cell epitope‐containing, non‐IgE‐reactive allergen fragments boost secondary IgE production in allergic patients 9. Furthermore, it has been shown that HIV‐infected patients with low CD4 cell counts continue to produce allergen‐specific IgE antibodies and that allergen‐specific IgE production can be boosted by allergen exposure in these patients 10.

In this study, we had the opportunity to investigate the effects of treatment with systemic cyclosporine A, a T‐cell‐targeting drug on allergen‐specific IgE production. Sera from patients with atopic dermatitis who had received systemic CyA treatment for up to 17 months were studied regarding IgE reactivity to exogenous, respiratory allergens and autoantigens.

## Materials and methods

### Characterization of patients and sera

**Figures 1 and 2:**
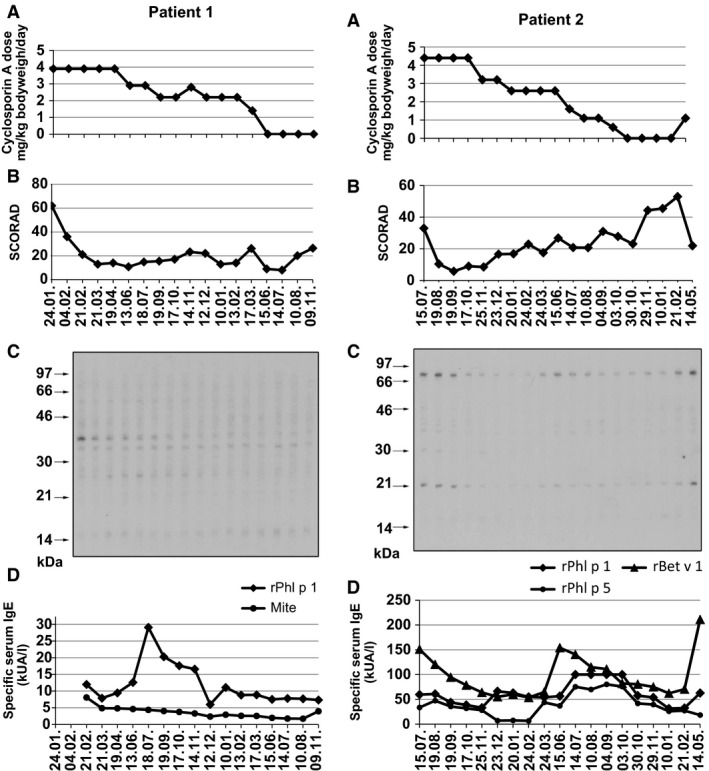
Time courses (x‐axes) of cyclosporin A treatment, skin symptoms, and IgE antibody reactivities to autoallergens and exogenous allergens in two AD patients. (A) Cyclosporin A dose (y‐axes: mg/kg bodyweight/day), (B) SCORAD documentation of skin manifestations (*y*‐axis: SCORAD indices), and (C) IgE autoreactivity to nitrocellulose‐blotted human epithelial cell extracts are displayed for different time points of blood sampling. Molecular weights (kDa) are shown at the left margin and (D) time course of specific serum IgE levels (y‐axes: kUA/L) to exogenous allergens (mite allergens, recombinant major birch pollen allergen, rBet v 1, and two recombinant major timothy grass pollen allergens, rPhl p 1 and rPhl p 5).

Residual serum samples from four atopic dermatitis patients, three males, one female, aged between 31 and 54, fulfilling the clinical and morphological criteria of AD 11 and atopic skin diathesis 12 were investigated in the study. These patients had participated in a study from 1993 to 1995 comparing the efficacy and tolerability of two CyA formulations: Sandimmun and Sandimmun Neoral, a micro‐emulsion of CyA with improved pharmacokinetic properties 13. In the course of this study, patients were treated systemically with CyA over time periods of 14–17 months. During the first 4 months, patients received doses between 3.7 and 4.4 mg/kg bodyweight per day which were then reduced and stopped after 11–13 further months (Figs 1 and S1, S2). Other treatments for AD were stopped 2 weeks before the onset of the CyA medication. The anonymous analysis of serum samples was approved by the local ethics committee. Quantitative measurements of allergen‐specific IgE antibodies (rBet v 1, rPhl p 1, rPhl p 5, mite allergens: *Dermatophagoides pteronyssinus*) were performed using the CAP FEIA system (Thermo Fisher, Uppsala, Sweden). Clinical staging was done according to the criteria of Hanifin and Rajka 11. Disease activity was estimated using the SCORAD as described 14.

### SDS‐PAGE, IgE immunoblotting

The human epithelial cell line A431 was obtained from the American Type Culture Collection (ATCC, Rockville, MD, USA). Cells were cultured and proteins extracted as described 15. Protein extracts were separated by preparative 12.5% SDS‐PAGE and blotted onto nitrocellulose membranes (Schleicher & Schuell, Dassel, Germany). Membrane strips were incubated with serum samples (1:10 dilution) that had been collected at different time points. Bound IgE antibodies were detected with ^125^I‐labeled antihuman IgE antibodies (RAST, Pharmacia Diagnostics) as described 16.

## Results

### Systemic CyA treatment improves skin symptoms in AD patients which is accompanied by a reduction in IgE autoreactivity

A strong improvement of skin symptoms as reflected by a reduction in the SCORAD was observed when patients received full‐dose CyA treatment during the first 4 months (Figs 1B, 2B and S1B, S2B). The reduction of the CyA dose and stopping of the treatment was accompanied by an increase in the SCORAD and deterioration of skin inflammation. The intensity of IgE autoreactivity to nitrocellulose‐blotted cell extracts seemed to reflect skin inflammation. For example, IgE autoreactivity to a 38‐kDa band recognized by patient 1 at the beginning of treatment was high when the SCORAD was high and disappeared for the remaining treatment period when the SCORAD was low (Fig. 1B, C). Similar observations were made for patients 2 and 3. IgE autoreactivity to a 70‐kDa and 21‐kDa band became weaker after treatment was started and appeared again when the SCORAD rose between March and September 1995 and in the end of the treatment in patient 2 (Fig. 2B, C). Also in patient 3, IgE autoreactivity to a 35‐kDa band disappeared when the SCORAD dropped and reappeared when it went up again (Fig. S1B, C). IgE autoreactivity in patient 4 was not very intensive but also became weaker after initiation of treatment (Fig. S2B, C).

### IgE levels specific for exogenous inhaled allergens are unaffected by CyA treatment

Strong increases of Bet v 1‐specific IgE levels were observed in the birch pollen allergic patient 2 shortly after the birch pollen seasons during the observation period (i.e., 24.03., 14.05.; Fig. 2D). Likewise, we found strong increases of grass pollen allergen (i.e., rPhl p 1, rPhl p 5)‐specific IgE levels in each of the grass pollen allergic patients after the respective grass pollen seasons (July–September) which then declined again (Figs 1D, 2D and S1D, S2D). The levels of IgE against house dust mite allergens, a perennial allergen source, were rather stable (e.g., patient 1: Fig. 1D) or showed some alterations (e.g., patients 3 and 4: Figs S1D and S2D). Changes of IgE levels to exogenous allergens thus seemed to be associated with allergen contact and were not associated with CyA treatment.

## Discussion

In this study, we analyzed IgE reactivity against endogenous and exogenous allergens in AD patients who had received systemic CyA treatment for up to 17 months 13. Systemic CyA has a strong immunosuppressive effect on T cells and therefore is used for the treatment of T‐cell‐mediated hypersensitivity diseases, transplant rejection, and graft‐versus‐host reactions 17. The systemic CyA treatment also led to a strong improvement of T‐cell‐mediated skin symptoms in the studied AD patients which was reflected by a strong drop of the SCORAD after treatment was initiated. When we compared skin symptoms and IgE autoreactivity, we noted that the reduction in skin inflammation was followed by a reduction in IgE autoreactivity which reappeared when the dose of CyA was reduced and skin symptoms worsened again. We have observed a similar association of IgE autoreactivity and skin inflammation earlier in another patient who was monitored regarding IgE autoreactivity over an extensive period 18. In this patient, we also noted that aggravation of skin inflammation was followed by an increase in IgE autoreactivity, whereas improvement of skin symptoms was followed by a decrease in IgE autoreactivity. We therefore think that tissue damage leads to the release of autoantigens which then triggers the production of autoreactive IgE antibodies and thus mirrors tissue damage. A reduction in skin inflammation by CyA treatment would thus lead to reduced release of autoantigens and thus explain the drop of IgE autoreactivity. However, also other mechanisms may influence IgE autoreactivity such as IL‐33‐mediated stimulation of pro‐inflammatory cytokines or skin inflammation induced by exposure to exogenous allergens (e.g., reappearance of IgE autoantibodies after birch pollen exposure in patient 2; time point 24. 03.; Fig. 2) 18, 19.

The kinetics and levels of IgE antibodies specific for environmental allergens under CyA treatment have not yet been investigated in any previous study and turned out to be different from the kinetics and levels of IgE autoantibodies. In fact, levels of birch and grass pollen allergen‐specific IgE followed allergen exposure. Pollen allergen‐specific IgE increased shortly after the pollen seasons and declined thereafter similar as observed for patients suffering from allergic rhinoconjunctivitis 7. Interestingly, increases of pollen allergen‐specific IgE levels were observed even when patients had received high doses of systemic CyA and T‐cell‐mediated skin inflammation had almost completely disappeared. A possible explanation for this finding could be that the boost of the secondary IgE production by pollen allergen exposure leads to a direct stimulation of IgE production by B cells without T‐cell help. The assumption that the boost of secondary IgE production may be independent of T‐cell help is in fact supported by other studies performed in different experimental setups 8, 9, 10. Collectively, these results indicate that it will be difficult to combat with secondary IgE production in already sensitized allergic patients with purely T‐cell‐targeting therapeutic strategies. In fact, also co‐stimulation blockade had no effects on IgE‐mediated asthma 20. We are well aware that the current study has many limitations such as the low number of patients and it is uncertain whether T‐cell help was indeed completely suppressed by CyA in the studied patients. Also, no information regarding symptoms of respiratory allergy was available for the patients. Nevertheless, our study shows that even high doses of CyA do not suppress allergen‐specific IgE production in humans.

## Funding

This work was supported by the Austrian Science Fund (FWF) grants P23350‐B11 and F4605.

## Author contributions

Acquisition of data was done by SL. PSG, DK, BW, RV, and BL contributed to conception and design of the study, analysis and interpretation of data, and drafting of the manuscript.

## Conflicts of interest

RV and BL have received research grants from the Austrian Science Fund (FWF). RV is consultant to Thermo Fisher Scientific, Uppsala, Sweden, Biomay AG, Vienna, Austria,and Fresenius Medical Care, Bad Homburg, Germany.

## Supporting information


**Figures S1 and S2.** Time courses of cyclosporin A treatment, skin symptoms, and IgE antibody reactivities to autoallergens and exogenous allergens in two AD patients. (A) Cyclosporin A dose (y‐axes: mg/kg bodyweight/day), (B) SCORAD documentation of skin manifestations (y‐axes: SCORAD indices), (C) IgE auto‐reactivity to nitrocellulose‐blotted human epithelial cell extracts are displayed for different time points of blood sampling. Molecular weights (kDa) are shown at the left margin and (D) time course of specific serum IgE levels (y‐axes: kUA/L) to exogenous allergens (mite allergens and two recombinant major timothy grass pollen allergens, rPhl p 1 and rPhl p 5). An arrow (“>“) indicates the time point of tissue damage due to massive sun exposure in patient 3.
