# Supplementary figures and images for: IgE responses to exogenous and endogenous allergens in atopic dermatitis patients under long‐term systemic cyclosporine A treatment
Source: Allergy. 2015 Sep 17;71(1):115–8. doi: 10.1111/all.12711 (PMC4950058; doi:10.1111/all.12711)

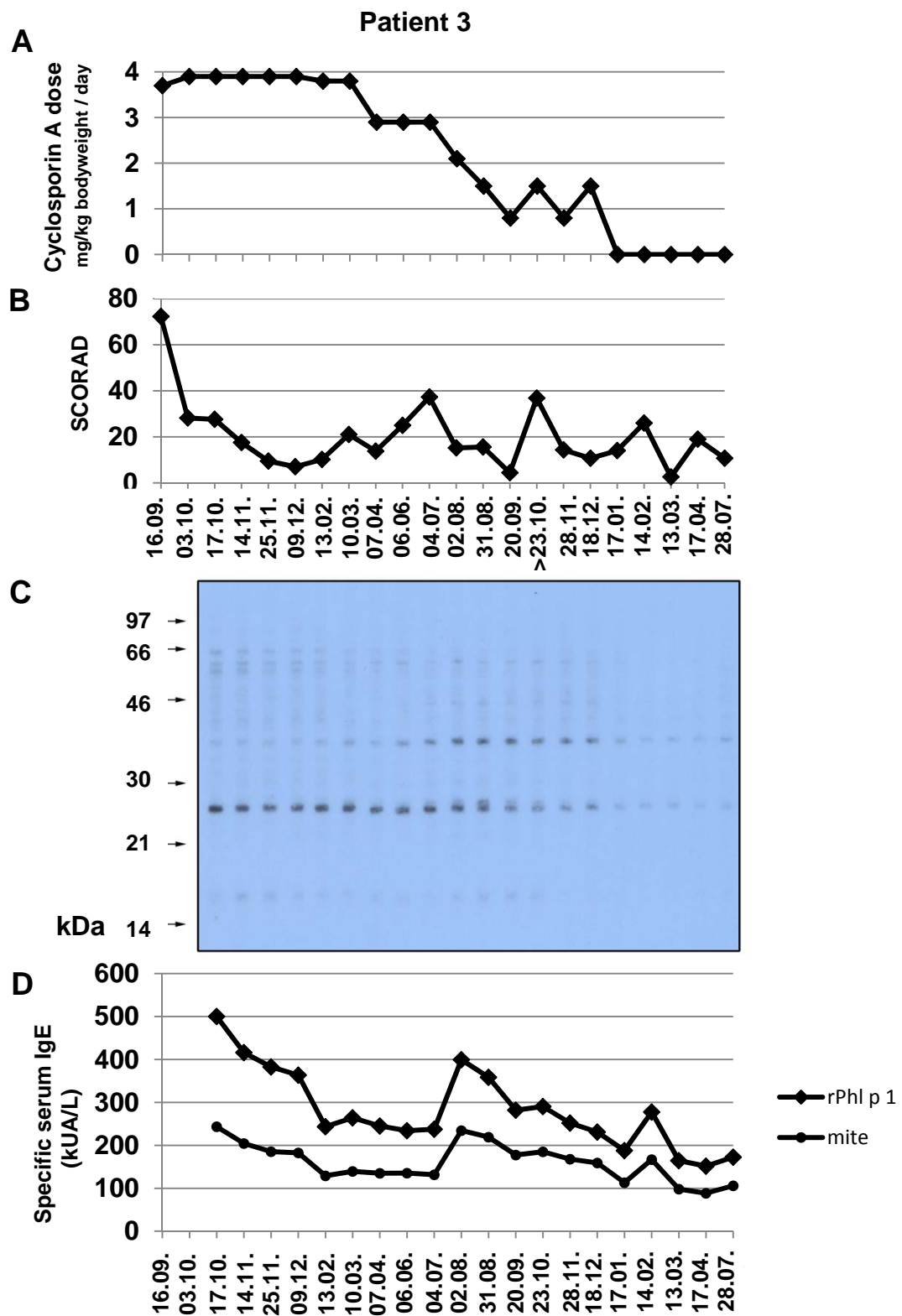

Supplemental Figure 1

Supplement: Supplementary file 1 — Figures S1 and S2. Time courses of cyclosporin A treatment, skin symptoms, and IgE antibody reactivities to autoallergens and exogenous allergens in two AD patients. (A) Cyclosporin A dose (y‐axes: mg/kg bodyweight/day), (B) SCORAD documentation of skin manifestations (y‐axes: SCORAD indices), (C) IgE auto‐reactivity to nitrocellulose‐blotted human epithelial cell extracts are displayed for different time points of blood sampling. Molecular weights (kDa) are shown at the left margin and (D) time course of specific serum IgE levels (y‐axes: kUA/L) to exogenous allergens (mite allergens and two recombinant major timothy grass pollen allergens, rPhl p 1 and rPhl p 5). An arrow (“>“) indicates the time point of tissue damage due to massive sun exposure in patient 3. [file ALL-71-115-s001.pdf]

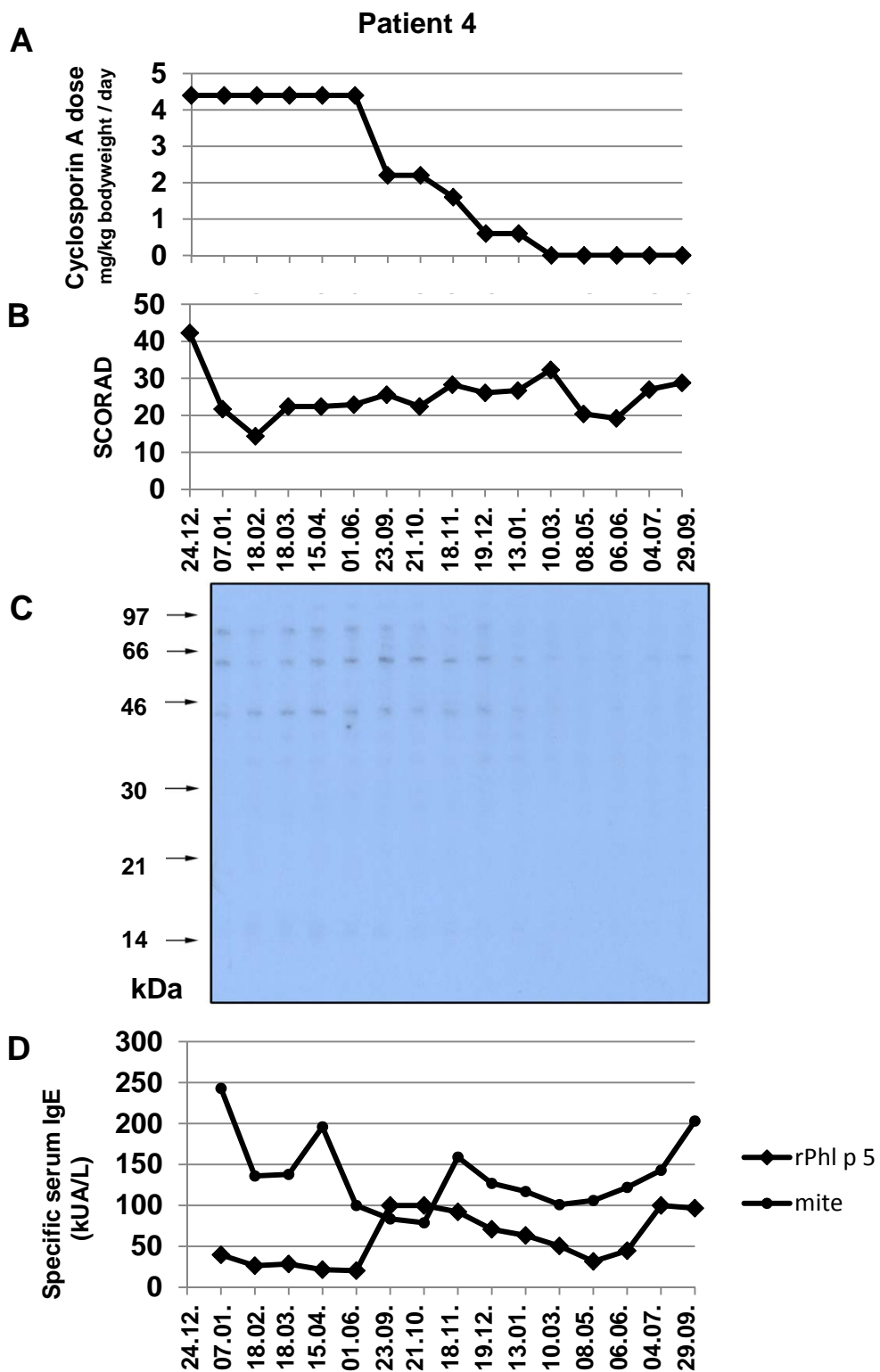

Supplemental Figure 2

Supplement: Supplementary file 2 [file ALL-71-115-s002.pdf]
